# Permanent Draft Genome Sequence of *Frankia* sp*.* Strain Allo2, a Salt-Tolerant Nitrogen-Fixing Actinobacterium Isolated from the Root Nodules of *Allocasuarina*

**DOI:** 10.1128/genomeA.00388-16

**Published:** 2016-05-19

**Authors:** Rediet Oshone, Mariama Ngom, Feseha Abebe-Akele, Stephen Simpson, Krystalynne Morris, Mame Ourèye Sy, Antony Champion, W. Kelley Thomas, Louis S. Tisa

**Affiliations:** a University of New Hampshire, Durham, New Hampshire, USA; b Laboratoire Mixte International Adaptation des Plantes et Microorganismes Associés aux Stress Environnementaux (LAPSE), Centre de Recherche de Bel Air, Dakar, Sénégal; c Département de Biologie Végétale, Laboratoire Campus de Biotechnologies Végétales, Faculté des Sciences et Techniques, Université Cheikh Anta Diop, Dakar-Fann, Sénégal; d Laboratoire Commun de Microbiologie IRD/ISRA/UCAD, Centre de Recherche de Bel Air, Dakar, Sénégal; e Institut de recherche pour le développement (IRD), UMR DIADE, Montpellier, France

## Abstract

*Frankia* sp. strain Allo2 is a member of *Frankia* lineage Ib, which is able to reinfect plants of the *Casuarinaceae* family, and exhibits a high level of salt tolerance compared to other isolates. Here, we report the 5.3-Mbp draft genome sequence of *Frankia* sp. strain Allo2 with a G+C content of 70.0% and 4,224 candidate protein-encoding genes.

## GENOME ANNOUNCEMENT

Salinization of soils and groundwater is a serious problem, especially in arid and semiarid lands, and has resulted in a drastic reduction in agricultural production (1). Worldwide, over 800 million hectares of land are affected (2). One potential solution for dealing with salt stress problems is reclaiming saline soils with fast-growing, multipurpose, salt-tolerant trees like the actinorhizal plants. Among the actinorhizal plants, the genus *Casuarina* grows well under these conditions and has been used in North Africa for these purposes. Actinorhizal plants form a nitrogen-fixing symbiosis with the genus *Frankia* that results in the ability of these plants to colonize harsh environments (3–5).

Based on phylogenetic markers, four major clusters are recognized within the genus (6–9), and genomes for representatives from each cluster have been sequenced (10–26). Cluster I contains two subclusters: one subcluster (cluster Ia) consists of *Frankia* spp. strains that associate with host plants in the *Betulaceae* and *Myricaceae* families, while the other subcluster (cluster Ib) is limited to *Casuarina* and *Allocasuarina* host plants*. Frankia* sp. strain Allo2 was isolated from root nodules of *Allocasuarina verticillata* (27). This strain showed an increased level of NaCl tolerance (R. Oshone and L. S. Tisa, unpublished data) and was sequenced to increase our understanding of salt-tolerance mechanisms and to provide insight about its interaction with actinorhizal plants. A comparative analysis of sequenced genomes among the salt-tolerant and salt-sensitive *Casuarina* strains may provide a clear picture of the role that these symbionts play in allowing these actinorhizal plants to colonize harsh environments, including saline soils.

The draft genome sequence of *Frankia* sp. strain Allo2 was generated at the Hubbard Genome Center (University of New Hampshire, Durham, NH, USA) using Illumina technology (28) techniques. A standard Illumina shotgun library was constructed and sequenced using the Illumina HiSeq2000 platform, which generated 26,647,886 reads (260-bp insert size), totaling 3,866.4 Mbp. The Illumina sequence data were assembled using CLC Genomics Workbench version 8.0.1 and AllPaths-LG version r41043 (29). The final draft assembly for *Frankia* sp. Allo2 consisted of 133 contigs in 110 scaffolds with an *N*_50_ contig size of 96.9 kb. The final assembled genome contained a total sequence of 5,352,211 bp with a G+C content of 70.0% and is based on 3,120.3 Mb of Illumina draft data, providing an average 583× coverage of the genome.

The assembled *Frankia* sp. strain Allo2 genome was annotated via the Integrated Microbial Genomes (IMG) platform developed by the Joint Genome Institute, Walnut Creek, CA, USA (30, 31) and resulted in 4,224 candidate protein-encoding genes and 45 tRNA and 3 rRNA regions.

### Nucleotide sequence accession numbers. 

This whole-genome shotgun project has been deposited at DDBJ/EMBL/GenBank under the accession number JPHT00000000. The version described in this paper is the first version, JPHT01000000.
